# Cumulative Cultural Evolution and Demography

**DOI:** 10.1371/journal.pone.0040989

**Published:** 2012-07-24

**Authors:** Krist Vaesen

**Affiliations:** School of Innovation Sciences, Eindhoven University of Technology, Eindhoven, The Netherlands; University of Oxford, United Kingdom

## Abstract

The idea that demographic change may spur or slow down technological change has become widely accepted among evolutionary archaeologists and anthropologists. Two models have been particularly influential in promoting this idea: a mathematical model by Joseph Henrich, developed to explain the Tasmanian loss of culture during the Holocene; and an agent-based adaptation thereof, devised by Powell et al. to explain the emergence of modern behaviour in the Late Pleistocene. However, the models in question make rather strong assumptions about the distribution of skills among social learners and about the selectivity of social learning strategies. Here I examine the behaviour of these models under more conservative and, on empirical and theoretical grounds, equally reasonable assumptions. I show that, some qualifications notwithstanding, Henrich’s model largely withstands my robustness tests. The model of Powell et al., in contrast, does not–a finding that warrants a fair amount of skepticism towards Powell et al.’s explanation of the Upper Paleolithic transition. More generally, my evaluation of the accounts of Henrich and of Powell et al. helpfully clarify which inferences their popular models do and not support.

## Introduction

Numerous recent publications [1]–[5] point out the dependence of cumulative cultural evolution on demography. Low population numbers, the thought is, may slow down or reverse processes of accumulation; high population levels, in contrast, spur cultural change. Justification for this thought is commonly derived from two highly influential studies, one by Henrich [6], the other by Powell and colleagues [7] (for other models linking demography and cultural adaptiveness, see [5], [8]–[11]). In the former, Henrich develops a mathematical model to account for the loss of tool complexity on Tasmania; the latter presents an agent-based model (based on Henrich’s mathematical model) to explain the Late Pleistocene appearance of modern human behaviour. From that it is concluded that the inaccuracies of cultural transmission will give rise to technological loss in case populations drop below a certain critical threshold (Henrich); and conversely, that these inaccuracies can be offset by increasing population size (Powell et al).

Bentley and O’Brien [12] suggest, however, that this result may be an artifact of two strong assumptions of both Henrich and Powell et al, namely: (A.1) that the distribution of skills in a population follow a Gumbel (or, in case of Henrich, a Logistic) rather than a Normal distribution; and (A.2) that social learners are strongly biased towards those mentors that are (extremely) skilled, rather than copying at random or following social cues (e.g., conformity, similarity). However, Bentley and O’Brien do not provide a real formal proof of their point. For that, one would need to adjust Henrich’s mathematical model and Powell et al’s agent-based model to observe whether the relation between population size and cumulation still holds when assumptions (A.1) and (A.2) are relaxed.

Below, I do precisely that. I first show that Henrich’s results, although becoming more fragile, still obtain when relaxing (A.1), i.e. under assumptions of Normality. Second, I demonstrate that the population effect in Powell et al’s model vanishes when (A.2) takes on more conservative values, i.e. when mentor selection is assumed random or conformist. The lack of firm theoretical and empirical justification for (A.2), I argue, seriously compromises Powell et al’s explanation of the Upper Paleolithic transition.

## Methods

### Henrich’s Model

#### Henrich’s original

Henrich’s mathematical model starts with a population of 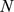 individuals, where each individual 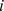 is skilled to an extent 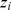 in a skill involving at least some culturally transmittable component. Through a process of inaccurate transmission, each new generation of 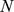 individuals acquires a 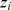 value from individuals of the previous generation. The average change in skill, 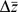, is given by the Price equation:
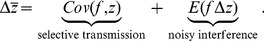
(1)


In this equation, the variable 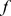 represents the likelihood that an individual will be copied. For instance, in case the 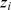 value of an individual 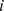 is high, and assuming that individuals are more likely to copy highly skilled individuals (i.e. individuals having a high 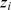 value), 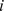 will be copied more frequently, and hence 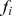 will be high.

As it stands, Equation (1) is still not very helpful for studying the specific conditions under which 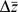 changes. For that purpose, Henrich makes a tractability assumption, namely that that all imitators copy the most skilled individual, 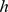 (having a 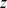 value 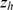). This is Henrich’s version of assumption (A.2) mentioned above: Henrich assumes extreme selectivity for the skill itself. Imitators are not just able to identify the most skilled individual 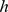, they are completely unaffected by context biases, such as biases of prestige, similarity, success or conformity.

So if all imitators copy the most skilled individual, 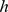, the likelihood of 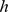 being copied (i.e. 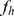) is 1, and the likelihood of other models being copied (i.e. 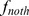) is 0. This tractability assumption simplifies matters considerably, because now the Price equation reduces to:
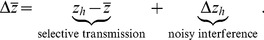
(2)


**Figure 1:**
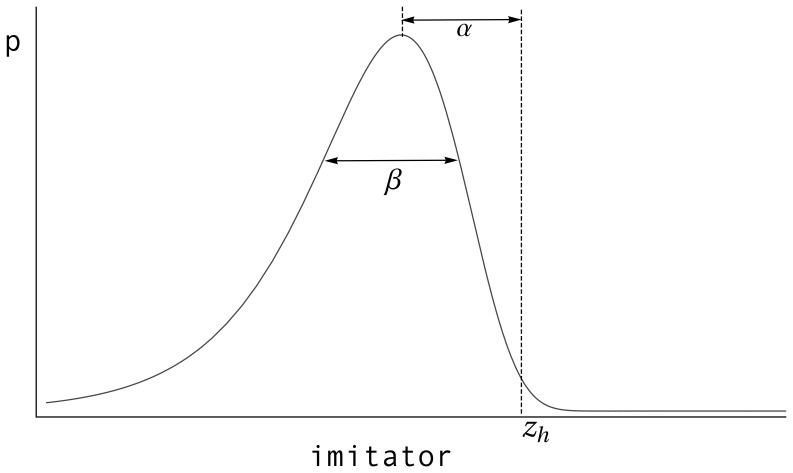
Gumbel probability density function for imperfect imitation.

The first part of Equation (2) takes the difference between the 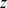 value of the subsequent generation (which equals 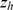, because all individuals are assumed to copy 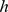) and the 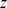 value of the earlier generation (which equals the average of 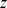
’s of that generation, i.e. 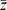). At this point, Henrich invokes assumption (A.1). He assumes that skills in the population are distributed according to a Gumbel distribution, so that the expected value of the highest values from a sample of size 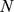 is given by
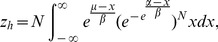
(3)which can be approximated by



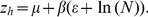
(4)Here 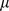 is the mode of the distribution, 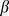 represents the distribution’s spread (see Figure 1), and 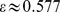 is the Euler-Gamma constant.

Still assuming a Gumbel distribution of skills, 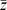 is given by
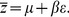
(5)


The second part of Equation (2), namely 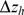, captures the transmission bias associated with copying 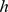. Each individual draws again from a Gumbel distribution (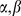) to determine her imitation error:
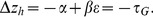
(6)


Since 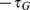 represents transmission inaccuracy, 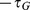 may be taken as a measure of skill complexity. In other words, a complex skill is a skill that is difficult to learn, and hence, one that is associated with high transmission inaccuracy 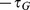. To be clear, other measures of skill complexity have been proposed. For the Gumbel, Henrich for instance uses the ratio 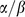 (rather than transmission inaccuracy, which for the Gumbel is 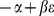), whereas for the Logistic (see below), he does work with transmission inaccuracy (which, in case of the Logistic is the mean 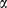). Powell et al [7] follow Henrich’s second approach even for their Gumbel-based implementation (so they take 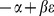 rather than 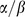 as a measure of skill complexity). Like Powell et al, I here thus follow Henrich’s second approach, first, because of its intuitive appeal, and second, because of at least one counter-intuitive feature of Henrich’s first approach (i.e. measuring complexity in terms of 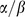). Consider, for instance, two skills, *A* and *B*, with corresponding 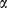
- and 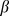-values, [9,9] for *A* and [0.1,0.1] for *B*. *A* and *B* have the same 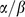-ratio. Still, plausibly *B* is much easier to imitate than *A*, as for *B* a much larger proportion of the population very (!) closely approximates the *z*-value of the model *h*. This fact is not captured by the ratio 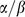, but *is* captured by 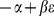 (−3.8 for *A* versus −0.042 for *B*).

Substituting Equations (4), (5) and (6) in Equation (2) yields
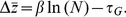
(7)


**Figure 2:**
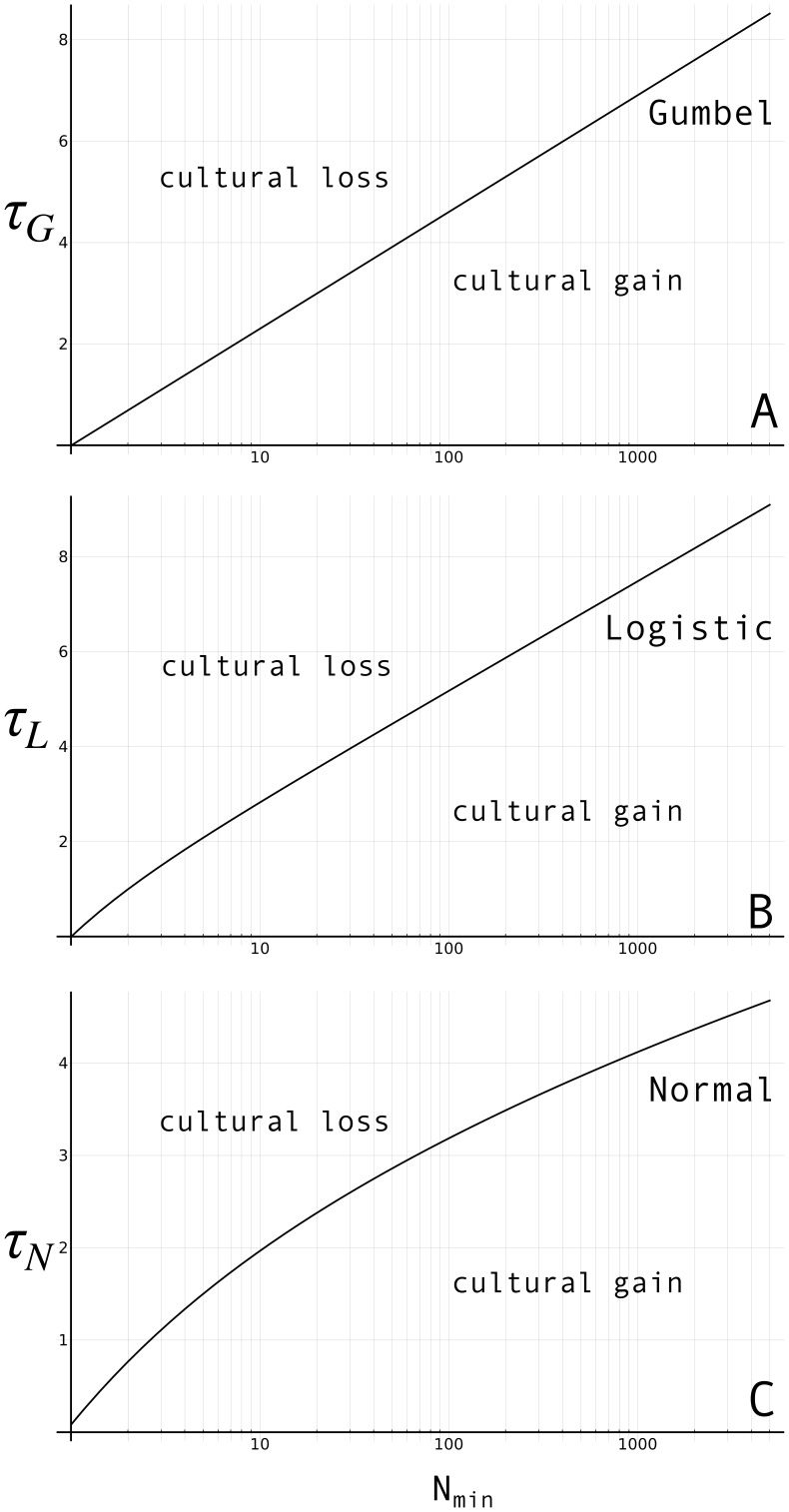
Critical population size (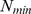) versus skill complexity (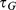, 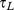, and 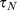) assuming a (A) Gumbel, (B) standard Logistic, (C) Normal distribution of skill levels. For the Gumbel, 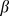 was set to 1, which corresponds to a variance 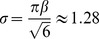. For the Normal curve, the same variance was used, i.e. 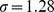.

Setting 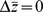 yields Figure 2A, with the X-axis representing the minimal population size needed for cumulation to occur, 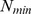. The area above the curve is associated with regimes of cultural loss (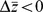), the area underneath it represents favourable conditions where skill accumulates (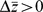). As one can see, indeed, population size has a considerable effect on the amount of skill complexity (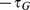) that can be maintained.

I must add here that Henrich also considers what happens in case skills follow a standard Logistic distribution. Following the same derivation as above, transmission inaccuracy for the Logistic, 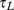, is according to Henrich (see his Appendix C) given by
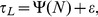
(8)where 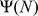 is the Digamma function at 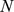. For 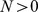, 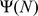 behaves nearly identically to 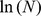. So when setting 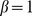 for the Gumbel (as done in Figure 2A), the Logistic curve is nearly identical to the Gumbel, only shifted up to an extent 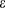, as can be seen in Figure 2B.

The two variants of the model indeed support the idea idea that only sizable populations of social learners are able to sustain processes of technological accumulation. To see whether this result is robust when we relax (A.1), the model now needs to be adjusted to assumptions of Normality.

#### Henrich’s model Normalized

Henrich provides no justification for using Logistic distributions. For the Gumbel, in contrast, his justification is that “a wide range of distributions, which includes the Gumbel distribution and the Normal distribution, yield the Gumbel distribution (approximately) when the extreme values are repeatedly sampled” [6] This may be true, it is not entirely satisfactory as justification. For although Henrich is right that one gets a Gumbel distribution both in case one repeatedly takes the highest value from a Normal distribution, and in case one repeatedly takes the highest value from a Gumbel distribution, the kind of Gumbel distribution one gets will differ (see [13]). So to really assess the impact of distribution choice, one cannot but just adjust Henrich’s model for assumptions of Normality.

To be clear, whether skills of the sort targeted by Henrich’s model (e.g., arrow-making, fishtrap-building) follow a Gumbel, Logistic or Normal distribution is an open empirical question. Gumbel distributions are typically used to account for extreme events (e.g., extraordinary hurricanes, floods, winds, earthquakes). Perhaps, intuitively, one may think that this matches cases where technological skills are taught by an extremely gifted tutor, say, a grandmaster training several novice archers. In such cases, at least when the population is considered as a whole (including tutors *and* novices), a Gumbel distribution doesn’t seem implausible at all, given the vast difference in skill between grandmaster and novices. Yet, in case we are interested in transmission across successive generations, what matters is not the skill distribution of the entire population, but the skill distribution only of the population of tutors (which, after learning, is “reproduced” by the generation of novices, who then become the generation of tutors). The idea that even among the generation of tutors (say, among all adult archers) such extreme differences exist, thereby justifying a Gumbel, is much less intuitive. Logistic assumptions seem more cautious; Normality assumptions at the very least not more controversial than the former two (but, for a note of skepticism, see [14])

How do we enter assumptions of Normality in Henrich’s model? We start with Equation (2) again, but redefine the variables at the right-hand side of the equation to reflect the idea that skill levels are distributed Normally. The expected value of the highest values drawn from a sample of size 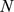, 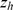, is now given by
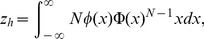
(9)where 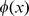 is the probability distribution function of Normal distributions, given by
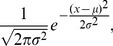
(10)and 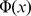 is the cumulative distribution function of Normal distributions, given by



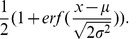
(11)Fortunately, Chen and Tyler [15] show that Equation (9) can be approximated with great accuracy by
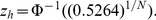
(12)


The other terms in Equation (2) are redefined as follows:
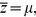
(13)

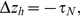
(14)with 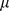 being the mean of the skills of the current population, and 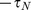 the transmission inaccuracy for the Normal. Equation (2) adjusted to normally distributed skill levels thus is given by



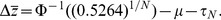
(15)Setting 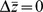 and 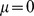, we get the curve in Figure 2C. Again, regimes of cultural loss are found above the curve, below it one finds regimes of cumulation.

### Powell et al’s Model

#### Powell et al’s original

Based on Henrich’s work, Powell et al [7] present an agent-based model to explain the appearance of modern behaviour in the Upper Paleolithic. They conclude that increases in population size and density (rather than increased cognitive capacity) well may account for that remarkable evolution in our lineage.

Inspired by Henrich, Powell et al assume: (A.1) Gumbel rather than Normal distributions of skills; and (A.2) a strong tendency to select the most skilled individuals in the population as mentors. In contrast to Henrich, however, Powell et al wish to explain an instance of cultural *gain*. Hence, in the absence of empirical evidence for (A.2)–evidence which Powell et al do *not* provide–a conservative strategy would not start from (A.2), but from assumptions working strongly *against* cumulation (e.g., random copying, conformity). Stated otherwise, to have a forceful demographic explanation one needs to show either that the population effect in Powell et al’s model survives such severe tests (i.e. assumptions of weak selectivity), or that early human social learning really exhibited the kind of strong selectivity assumed in the model. Let me first examine the first option, that is, how Powell et al’s model fares under conditions of weak selectivity.

The model of Powell and colleagues contains a set of agents that undergo both vertical and oblique transmission. In particular, in each generation the model goes through the following steps:

1. *Vertical transmission*: each agent behaves as a cultural parent, producing one cultural offspring. The offspring individual takes the 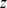-value of the parent according to the transmission process described by Henrich.
2. *Oblique transmission*: each offspring is given the opportunity to undergo oblique transmission, by selecting an oblique model from only those adults with 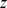-values greater than that they received from their own parent, with *probability proportional to the magnitude of the 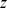-value difference*. In the absence of such qualified oblique model, this step is skipped. The offspring individual takes the 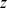-value of the oblique model, again according to the transmission process described by Henrich, but only if it exceeds its current 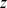-value (i.e. the one obtained from the parent).
3. *Replacement*: the offspring generation replaces the parent generation, and the average skill level of the population, 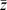, is measured.

At this point, two things need to be stressed. First, Powell et al’s original model contains also a fourth step, in which agents are given the opportunity to migrate from one subpopulation to the other. But since the authors find that migratory activity among a set of subpopulations has the same effect on skill accumulation as increasing the size of a single isolated population, this step is ignored in the remainder. That is, my adjusted model will only concern isolated populations with varying sizes 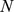.

Second, the strong selectivity assumption (A.2) is made in step 2, in the passage in italics: adults choose mentors proportionally to their skill. This is a somewhat weaker form of selectivity than that assumed by Henrich in Equation (7), but still much stronger than the two I will consider, namely random copying and conformity.

#### Powell et al’s model adjusted to random copying and conformity

In the random copying condition, offspring select an oblique model at random and adopt the latter’s 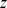-value (through Henrich’s transmission mechanism) just in case it exceeds the 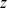-value received from the parent. Merely in virtue of the distribution of skills, random selection will automatically favor 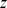-values closer to the mode of the distribution.

Such randomness may reflect two things. First, it may capture the fact that, especially in larger populations and/or populations with low cultural interconnectedness, not all cultural parents are available to all to learn from, so that happenstance decides which parents are assigned to whom. Second, the random copying condition may imply absence of learning biases or, if present, any systematic expression of them.

As Bentley and colleagues remark [16], random copying is a suitable null hypothesis for examining the conditions of cultural change. This holds especially for the study of Powell and colleagues, whose aim it is to demonstrate that demographic factors, *without any change in cognitive machinery*, could have caused the emergence of modern behaviour in the Upper Paleolithic. After all, the strong pay-off bias assumed by Powell et al may be one of those cognitive inventions which the authors wish to prove the dispensability of, perhaps involving mechanisms for evaluating pay-off differences or prestige and for inhibiting present drives until a better-than-the-present mentor is found.

Moreover, Powell et al’s selectivity is at odds with the conformity biases widely discussed in studies of cultural evolution. Conformity here refers to the propensity of individuals to *actively* scout the population for the most common behaviour. Such conformity, now, is my second adjustment to the Powell model, and is implemented in two ways (as in [17]). In Conformity #1, offspring randomly select an oblique model with a 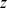-value in the interval 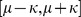, with 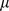 being the mode of the distribution. With 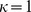 and 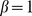, the interval amounts to four times the spread 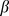 of the Gumbel, which corresponds to a fairly modest form of conformity, including values already quite remote from the mode. Results so obtained would *a fortiori* obtain in case conformity is stronger. In Conformity #2, offspring select an oblique model with probability inversely proportional to the magnitude of the difference between the model’s 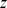-value and the mode of the distribution.

For given values of 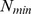, I simulated widely over 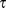, to find the level of complexity that could be sustained by a population of critical size 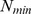. Simulations were performed for four learning strategies: random copying, conformity and the direct biases of Henrich and of Powell and colleagues. I did so both assuming Gumbel and Normal distributions of skills. For more details about the implementation of the model, I refer the reader to Text S1.

## Results

### Henrich’s model

#### Result 1: Henrich’s qualitative results still obtain under assumptions of Normality

As can be seen in Figure 2, the Normalized model largely reproduces the qualitative results of Henrich’s Gumbel and Logistic implementations: at lower 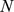
’s, decreases of 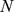 have a high impact on the maximum sustainable level of skill complexity (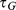, 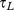 and 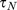 respectively); for higher 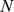
’s, the population effect is weaker. As such, the Normalized model is in line with Henrich’s demographic explanation of the Tasmanian loss of culture during the Holocene: a reduction of the Tasmanian population from 5,000 to 2,000 (Henrich’s estimates) yields a reduction of skill complexity, also under assumptions of Normality.

#### Result 2: Still, Normal populations are at a somewhat lower risk of cultural loss due to population reduction

The above may seem to prove wrong Bentley and O’Brien [12]: since they also hold under assumptions of Normality, Henrich’s results do not seem an artifact of his specific assumptions regarding skill distributions. Yet, a closer comparison of the Gumbel/Logistic and Normal models *does* reveal a subtle difference–much in line with Bentley and O’Brien’s predictions.

**Figure 3:**
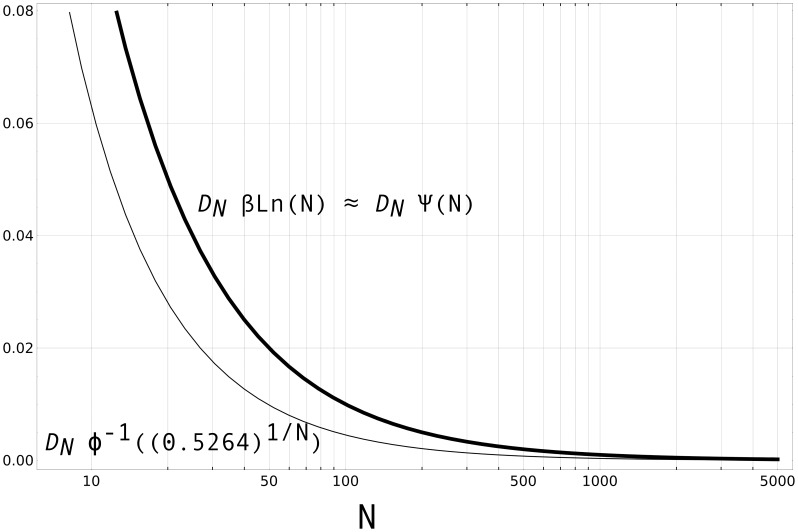
Comparison of the sensitivity of Gumbel/Logistic versus Normal populations to cultural loss due to population reduction. Comparison is done by plotting the derivative functions of, on one hand, 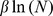 and 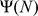, and of 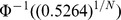 on the other. For the first 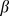 was set to 1; correspondingly, for the latter 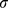 was set to 1.28.

More specifically, Normal populations are less susceptible to loss due to population reductions than Gumbel/Logistic populations. To appreciate this, one just needs to compare the derivatives of the functions governing the Gumbel/Logistic and Normal curves presented in Figure 2. From Equations (7), (8) and (15), we learn that these functions are 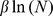 (for the Gumbel), 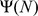 (for the Logistic), and 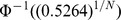 (for the Normal). Their derivative functions are plotted in Figure 3. The derivative function of the Normal (i.e. 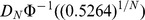 is below the derivative function of the Gumbel/Logistic (i.e. 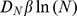 and 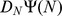), for *all* values of 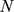 (even though for larger 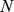
’s, the difference is indiscernible in the figure). This implies that changes in 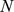 correspond *generally* to smaller changes in the dependent output (transmission inaccuracy, in our case) for Normal than for Gumbel/Logistic populations. Also, above 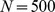, the derivative function of the Normal is constant at close to 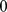. So, as long as they do not drop below a certain threshold (around 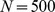), Normal populations will suffer only marginally from reductions in size. The derivative function of the Gumbel/Logistic, in contrast, bottoms out slower (for instance, the sensitivity to population changes of the Normal at 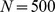 is reached only at around 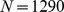 in the Gumbel/Logistic) and always remains slightly above the derivative of the Normal.

To stress again, these differences are subtle, and arguably insufficient to undermine Henrich’s explanation of the loss of Tasmanian culture (although the matter is impossible to settle empirically). Yet, what stands out is that demographic explanations under assumptions of Normality are somewhat more demanding: to account for an observed loss of culture, bigger population reductions must be assumed.

#### Result 3: Henrich’s results do not extrapolate to instances of cultural gain, especially not under assumptions of Normality

Henrich’s model was devised explicitly to address instances of cultural *loss*. Therefore it assumes, per (A.2), perfect mentor selection; loss demonstrated under these conditions makes loss even more likely in cases of weaker selectivity. (A.2) is thus highly conservative and, for Henrich’s purposes, properly so.

However, in explanations of cultural *gain*, the opposite holds. Instead of (A.2), conservative assumptions are now those that assume much-less-than-perfect mentor selection, such as copying at random, or selection on the basis of social cues (conformity in particular). Cumulation demonstrated under such severe conditions makes cumulation even more likely in case learning biases do not work so strongly against it.

Hence, without modifying its assumptions (i.e. assuming much-less-than-perfect mentor selection), Henrich’s model is likely to overestimate the cultural gain resulting from increases in population size (unless, of course, the assumption of perfect mentor selection really would conform with empirical observations). Importantly, Henrich himself doesn’t use his model for that purpose (in contrast to Powell et al, see below). Moreover, he presents an adjustment of Equation (7) (see his Equation 4) which can be used to examine the effects of random copying (although it is presented as an adjustment to accommodate vertical transmission). In case all individuals copy at random, the model now predicts no cumulation, which is more or less in line with the results presented in the next section. It is unclear how Henrich’s model would behave under conditions of strong conformity, but these will be taken up in the next section.

The considerations above apply irrespective of distribution choice: neither the Gumbel/Logistic nor the Normal implementation of Henrich’s model are duly conservative when it concerns demographic explanations of instances of technological gain. But, the Normalized model is even worse in this respect. For, given the more modest population effect under assumptions of Normality (see result b), Normal populations will even more readily exhibit cultural stasis, despite increases of 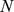. The same slight deviation from perfect mentor selection may yield stasis in Normal populations, merely slowed down cumulation in Gumbel/Logistic populations.

### Powell et al’s Model

#### Result 1: Powell et al’s results do not obtain under conditions of weak selectivity

**Figure 4:**
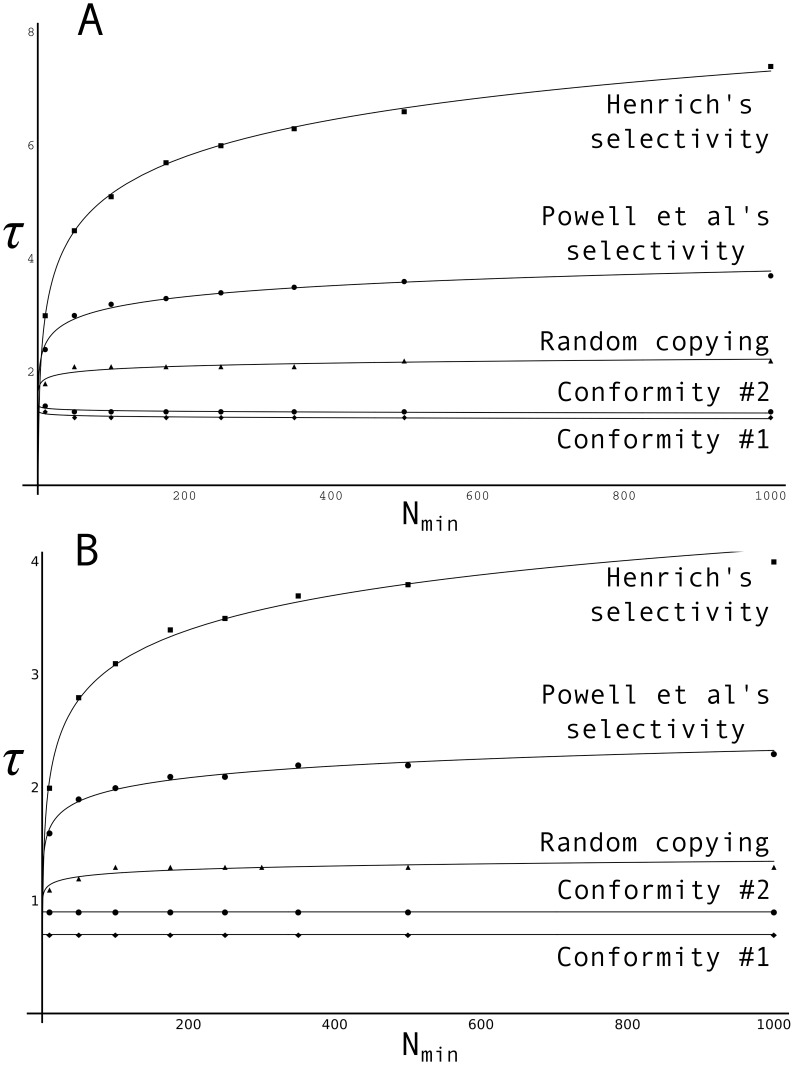
Critical population size (N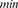) versus skill complexity (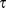) for different selection biases, assuming (A) a Gumbel distribution of skills; and (B) a Normal distribution of skills. For both (A) and (B), 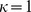. (A) corresponds to Figure 4B of Powell et al [7]. Note that doubling kappa, i.e. 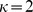, does not change the qualitative results; now the curve is constant at 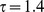 for the Gumbel, and at 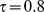 for the Normal.

Figure 4 presents simulated data for the four learning biases, assuming Gumbel (Fig. 4A) and Normal (Fig. 4B) distributions of skills respectively. The first thing to note is that the dependence of cumulation on population size is virtually absent in case of random copying (some population effect persists for very small populations, i.e. 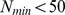), and completely absent in case of conformity (both Conformity #1 and Conformity #2). This places a major burden of proof on Powell and colleagues. Their claim that demographic change rather than increased cognitive capacity caused the emergence of modern behaviour is credible only to the extent that they can show Upper Paleolithic social learning to have been pay-off based, rather than random or conformist. What evidence is there in support of this claim?

There are indeed theoretical reasons for assuming pay-off biases [18], [19] and, in laboratory settings, Western subjects appear to be generally inclined to imitate successful individuals (see the review by Mesoudi [20]; more recently, see [21], [22]). But, there is also good theoretical and empirical evidence for conformist transmission: models of Henrich and Boyd [23] suggest that conformist biases are likely to evolve whenever social learning evolves; Henrich [24] finds evidence of conformist transmission in field data on the diffusion of innovations; Henrich and Boyd [25] and Mesoudi [20] review a whole set of empirical studies demonstrating the powerful human propensity to conform. Importantly, it is uncertain whether the empirical findings (both with respect to pay-off and conformist biases) extend to non-Western small-scale societies. Although Henrich and Broesch [26] provide evidence for prestige biases among Fijian villagers, no corresponding evidence is available for contemporary hunter-gatherers [27].

In light of the above, I think the most honest thing to say is that the issue is undecided: there is insufficient warrant for choosing either bias (pay-off or conformist) as being the most dominant in human social learning, let alone as being the most dominant in Upper Paleolithic cultural learning. Hence, Powell et al have little warrant for focusing only on the most optimistic scenario (strong pay-off biased transmission), simply ignoring the more conservative one (strong conformist transmission).

Second, what about the random copying condition? On the first interpretation of the condition, randomness has nothing to do with the psychological make-up of the social learners in question, but with the fact that external factors often do the selection for us, for instance, when populations are big and/or exhibit low cultural interconnectedness. Chance effects due to population size are presumably limited for the sizes assumed by Powell et al (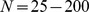); effects due to cultural interconnectedness, in contrast, may be much more real. Given the costs associated with acting as a mentor, there are strong theoretical reasons and there is at least suggestive empirical evidence in support of the idea that most transmission of early human crafts happened among kin–i.e. predominantly vertically, and when obliquely, just within the extended family [28], [29]–resulting in low cultural interconnectedness. Now if oblique transmission in the Upper Paleolithic occurred only within the extended family indeed, assumptions of randomness seem justifiable; whether a social learner will be given a decent opportunity for learning obliquely depends on the contingent fact of being born or not being born in a family containing one or more highly skilled oblique models.

According to the second interpretation, the random copying condition implies randomness in the strategies of social learners when selecting a mentor. The learner is assumed either to really select mentors at random (i.e. she is insensitive to clues about such things as the mentor’s success or the popularity of the mentor’s behaviour), or to switch strategies seemingly at random (e.g, conformist under time pressure, pay-off based otherwise; pay-off based if cheap, conformist otherwise; conformist in the production of threaded shell beads and tattoo kits, pay-off based in matters of blades and burins). While there is accumulated evidence that contemporary humans do not just select mentors at random (for a recent report, see [22]), and thus indeed *are* responsive to the features of the mentors they choose, little is known about the precise conditions under which they are responsive to which clues, that is, about when which bias can be singled out as being dominant. Given the aforementioned uncertainties regarding pay-off and conformity biases in the Upper-Paleolithic in particular, it definitely makes sense to consider what happens in case no systematic trends in Upper-Paleolithic learning biases are assumed. The random copying condition acknowledges that we are unsure about what would qualify as a more realistic one.

In sum, given that Powell et al’s results do not survive assumptions of weak selectivity, the authors need to provide a justification for the strong pay-off biases they assume. In light of the evidence just discussed, this will be a difficult, if not impossible, task.

#### Result 2: Even given strong pay-off biases, the population effect vanishes at higher *N*’s

As can be seen in Figures 4A and 4B, once a certain population size is reached, population size has little to no effect *even under a regime of selectivity à la Powell et al*. For values of 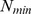 above 250, nothing much is gained by adding more members to the population; effective populations of 250 and 1,000 are able to sustain largely the same levels of skill complexity 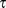. This is an important qualification: according to the model of Powell et al, effects of demography only play in effective populations of fairly limited size (so that it becomes crucial to establish that Upper Paleolithic populations were indeed so limited in size). The model, in contrast to economic models of technological growth (see [30], and references therein), does not warrant inferences about the existence of a *general* population effect, which holds for small and large populations alike.

### Conclusion

In this paper, I have examined the robustness and explanatory power of two very influential models linking demography and cumulative culture, a mathematical model by Henrich, and an agent-based implementation thereof by Powell and colleagues. In particular, I have tested Bentley and O’Brien’s suggestion that the results obtained by Henrich and by Powell et al may be an artifact of two fairly strong assumptions: (A.1) that cultural skill levels follow a Gumbel (or a Logistic, in case of Henrich) rather than a Normal distribution; and (A.2) that social learning biases are strongly pay-off based. Relaxing (A.1) and (A.2), I found, has limited impact on Henrich’s account, but seriously comprises that of Powell et al.

More specifically, Henrich’s model still exhibits a population effect if (A.1) is relaxed, although adaptive culture in Normal populations appears generally less sensitive to population reduction than adaptive culture in Gumbel/Logistic populations. Relaxing (A.2), as anticipated by Henrich himself, potentially removes the effect completely. But since Henrich’s aim is to explain an instance of cultural loss, (A.2), even if unrealistic, provides the severest test possible. Loss demonstrated under (A.2) would be even more likely in case learning biases were less pay-off based.

Powell et al’s model, in contrast, targets an instance of cultural gain. Here severe tests are those in which (A.2) *is* seriously relaxed. Under such severe conditions, I have shown, Powell et al’s model predicts cultural stasis rather than cumulation. This finding, together with the absence of strong evidence in support of (A.2), seriously weakens Powell et al’s case for a demographic explanation of the Upper Paleolithic transition.

The present study is limited in scope in that it has focused on just two, even if very influential, models. Future research should determine whether alternative models linking population size and cultural adaptiveness [5], [8]–[11] are (also) causes for concern. But I hope that this paper, at the very least, has demonstrated the usefulness of such robustness tests: these help to sort out the claims we are most interested in, to wit, claims that hold independently from the simplifying assumptions of the models they are based on.

## Supporting Information

Text S1 Details of the implementation of the simulations.
(PDF)
