# Supplementary material for: Cumulative Cultural Evolution and Demography
Source: PLoS One. 2012 Jul 24;7(7):e40989. doi: 10.1371/journal.pone.0040989 (PMC3404092; doi:10.1371/journal.pone.0040989)
Supplement: Text S1 — Details of the implementation of the simulations. (PDF) [file pone.0040989.s001.pdf]

# Supporting Information File S1 - Details of the implementation of the simulations

Simulations for the second part of the paper were implemented in NetLogo. Simulations assume a population of $N = 10, 50, 100, 175, 250, 350, 500, 1000$ agents. Each individual has a skill level that measures the individual's ability at some culturally inherited skill. In each run, models go through three stages: vertical transmission, oblique transmission, and replacement.

During vertical transmission, offspring receive their $z$-value from their parent. In particular the offspring's $z$-value is given by the parent's $z$-value, minus the structural transmission inaccuracy $\tau$, plus an individual error, randomly drawn from a Gumbel[0;1]-distribution or Normal[0;1.28]-distribution.

During oblique transmission, offspring individuals are given the opportunity to learn from one other adult. The adults they select as mentors differs for each of the four learning biases mentioned in the main text:

1. in case of *Henrich's selectivity*, offspring select the most skilled individual of the population;
2. in case of *Powell et al's selectivity*, offspring select an adult from only those adults with $z$-values greater than that they received from their own parent, with probability proportional to the magnitude of the $z$-value difference;
3. in case of *random copying*, offspring select a mentor at random;
4. in case of *Conformity #1*, offspring randomly select a mentor from those adults whose skill level is in the range $[\mu - \kappa, \mu + \kappa]$; and
5. in case of *Conformity #2*, offspring select an oblique model with probability inversely proportional to the magnitude of the difference between the model's $z$-value and the mode of the distribution.

Offspring then receive their $z$-value from the selected mentor. In particular the offspring's $z$-value is given by the $z$-value of the oblique model, minus the structural transmission inaccuracy $\tau$, plus an individual error, randomly drawn from a Gumbel[0;1]-distribution or Normal[0;1.28]-distribution. In case the result does not exceed the $z$-value received from the parent, the offspring individual keeps the $z$-value received from the parent.

Finally, during replacement, the offspring generation replaces the parent generation.

Models go through 100 runs, after which the overall change in average skill, $\Delta\overline{z}$, is measured. To account for stochastic variation in simulation outcomes, 100 iterations were performed and results were averaged across these.
